# MRI characteristics in acute ischemic stroke patients with preceding direct oral anticoagulant therapy as compared to vitamin K antagonists

**DOI:** 10.1186/s12883-020-01678-4

**Published:** 2020-03-11

**Authors:** Thomas Raphael Meinel, Johannes Kaesmacher, Jan Gralla, David J. Seiffge, Elias Auer, Sebastién Frey, Marwan El-Koussy, Marcel Arnold, Urs Fischer, Martina Göldlin, Simon Jung, Arsany Hakim

**Affiliations:** 1 Department of Neurology, Inselspital, Bern University Hospital, University of Bern, Freiburgstrasse 8, CH-3010 Bern, Switzerland; 2 Institute of Diagnostic and Interventional Neuroradiology, Institute of Diagnostic, Interventional and Pediatric Radiology and Department of Neurology, University Hospital Bern, Inselspital, University of Bern, Bern, Switzerland; 3 University Institute of Diagnostic and Interventional Neuroradiology, Inselspital, Bern University Hospital, University of Bern, Bern, Switzerland

**Keywords:** Acute ischemic stroke, DOAC, VKA, Large vessel occlusion, Anticoagulation, Infarction size, Hemorrhagic transformation

## Abstract

**Background:**

Despite the utility of neuroimaging in the diagnostic and therapeutic management of patients with acute ischemic stroke (AIS), imaging characteristics in patients with preceding direct oral anticoagulants (DOAC) compared to vitamin K antagonists (VKA) have hardly been described. We aimed to determine presence of large vessel occlusion (LVO), thrombus length, infarction diameter, and occurrence of hemorrhagic transformation in AIS patients with preceding DOAC as compared to VKA therapy.

**Methods:**

Using a prospectively collected cohort of AIS patients, we performed univariate and multivariable regression analyses regarding imaging outcomes. Additionally, we provide a sensitivity analysis for the subgroup of patients with confirmed therapeutic anticoagulation.

**Results:**

We included AIS in patients with preceding DOAC (*N* = 75) and VKA (*N* = 61) therapy, median age 79 (IQR 70–83), 39% female. Presence of any LVO between DOAC and VKA patients (29.3% versus 37.7%, *P* = 0.361), and target LVO for endovascular therapy (26.7% versus 27.9%, *P* = 1.0) was equal with a similar occlusion pattern. DOAC as compared to VKA were associated with a similar rate of target LVO for EVT (aOR 0.835, 95% CI 0.368–1.898). The presence of multiple lesions and characteristics of the thrombus were similar in DOAC and VKA patients. Acute ischemic lesion diameter in real world patients was equal in patients taking DOAC as compared to VKA. Lesion diameter in VKA patients (median 13 mm, IQR 6–26 versus median 20 mm, IQR 7–36, *P* = 0.001), but not DOAC patients was smaller in the setting of confirmed therapeutic VKA. The frequency of radiological hemorrhagic transformation and symptomatic intracranial hemorrhage in OAC patients was low. Sensitivity analysis considering only patients with confirmed therapeutic anticoagulation did not change any of the results.

**Conclusion:**

Preceding DOAC treatment showed equal rates of LVO and infarct size as compared to VKA in AIS patients. This study adds to the knowledge of imaging findings in AIS patients with preceding anticoagulation.

## Background

Patients with atrial fibrillation (AF) have a five-fold increased risk of acute ischemic stroke (AIS). Therefore, oral anticoagulation (OAC) is recommended after risk stratification to prevent thromboembolic events [1]. Due to the increased risk of intracerebral hemorrhage and downsides of management of Vitamin K antagonists (VKA), direct oral anticoagulants (DOAC) are now recommended as first line OAC in patients with AF [1, 2]. Even with the pronounced preventive reduction of AIS by OAC [1], AIS occurs with an estimated rate of about 1–2% per year in patients taking a DOAC [3].

Initial imaging in AIS is crucial to rapidly obtain an accurate diagnosis, select patients for acute recanalization treatments, predict outcome, guide etiological work-up and initiate early secondary prevention [4–6]. Nevertheless, data on imaging characteristics of AIS in patients taking OAC, especially DOAC are sparse [7, 8]. Few comparative small studies looked at size, volume and presence of a large vessel occlusion (LVO) [9, 10] in patients taking DOAC versus VKA, with preliminary results in favor of DOAC. However, no laboratory assessment of OAC activity or information on compliance for DOAC was available.

Hence, we aimed to explore the frequency of any LVO or target LVO for endovascular stroke treatment and other imaging characteristics of AIS in a prospectively collected registry of patients admitted to our comprehensive university stroke center prescribed with OAC. Additionally, we present a sensitivity analysis considering only patients with confirmed therapeutic DOAC and VKA activity on admission.

## Methods

Inclusion criteria for this study were a diagnosis of AIS and preceding OAC prescription at the time point of symptom onset (either DOAC or VKA). We selected a random sample of patients fulfilling the inclusion criteria from our prospectively consecutive local stroke registry from February 2015 to August 2017. We chose random sampling because of lack of capacity to analyze the images of all DOAC and VKA patients of the registry. For this purpose, a random number between 0 and 1 was generated using the excel function = RAND for each patient. By sorting the random number ascendingly, the first 80 patients were included in the study. We analyzed only patients with MRI on admission, which represents the preferred imaging modality in our center (about 60–70%). We excluded patients with transient ischemic attacks, stroke mimics, non-cerebral ischemic events, refusal of further use of biological data and missing information on OAC medication from this analysis. Antiplatelet prescription did not affect group assignment. For the sensitivity analysis, we further defined confirmed therapeutic OAC as specific drug activity > 50 ng/ml in patients taking DOAC [11–15] and INR > 1.7 in VKA patients. If no specific drug activity was available in patients taking DOAC, confirmed therapeutic OAC was defined as reported previously [16].

We assessed the following information from the registry and in case of missing items in the medical records: demographic variables (age, sex, prestroke modified Rankin Scale (mRS)), cardiovascular risk factors, clinical parameters (blood pressure, National Institutes of Health Stroke Scale (NIHSS), onset type, TOAST-etiology, antithrombotic, antihypertensive and lipid lowering medication before admission, laboratory parameters (cholesterol, glucose, creatinine, and international normalized ratio INR) and acute recanalization treatment such as intravenous thrombolysis (IVT) and endovascular therapy (EVT).

### Imaging protocol

We acquired MRI scans on the day of admission, either on a 1.5 T scanner (Siemens Magnetom Avanto and Siemens Magnetom Aera, Siemens Medical Solutions, Erlangen, Germany) or a 3 T system (Siemens Magnetom Verio, Siemens Medical Solutions, Erlangen, Germany). Our MRI protocol includes axial diffusion-weighted imaging (DWI) with apparent diffusion coefficient (ADC) (5 mm slice thickness), Fluid-attenuated inversion recovery (FLAIR) (5 mm slice thickness), susceptibility-weighted imaging (SWI) (1.6 mm slice thickness), and a time of flight (TOF) angiography (0.5 mm slice thickness). After application of intravenous Gadobutrol (Gadovist; Bayer Schering Pharma, Berlin, Germany) in an antecubital vein with a 5 ml/s injection rate, we acquired a standard dynamic susceptibility contrast (DSC) MRI perfusion (Tmax, 5 mm slice thickness) as well as a contrast enhanced T1-weighted sequence (slice thickness 5 mm). Finally, a contrast enhanced magnetic resonance angiography (CE MRA) of the head and neck vessels was acquired after injection of a second bolus of Gadobutrol with a 3 ml/s injection rate. Post-processing of DSC was performed using Olea sphere v.2.3 (Olea Medical, La Ciotat, France), using deconvolution method with oscillant singular value decomposition. Follow-up imaging was performed using the same scanners and same MRI protocol.

### Outcomes and imaging analysis

Two raters (EA and SF) assessed the imaging outcomes after a standardized training and blinded to patients’ OAC status. All ratings were controlled by a board-certified neuroradiologist (AH) with more than 10 years’ experience in stroke imaging. The primary outcome was the presence of any LVO defined as occlusion of an artery supplying the brain visible on TOF or contrast enhanced MR-angiography. Secondary outcomes included: target LVO for EVT defined as LVO until or proximal to A1/M2-segment of the anterior circulation or P1-segment of the posterior circulation; presence and length of thrombus as determined by SWI; size of hypoperfusion; degree of DWI infarction core and hypoperfused area mismatch defined as small mismatch (infarction core ≥2/3 of the hypoperfused region), moderate mismatch (infarction core > 1/3 and < 2/3 of the hypoperfused region) and large mismatch (infarction core ≤1/3 of the hypoperfused region) by visual judgment; maximal diameter in mm of the acute ischemic lesion as determined in DWI (axis 1), maximal diameter of a perpendicular axis of the acute ischemic lesion (axis 2). In case of multiple lesions, the largest lesion was analyzed for the primary analysis. Other outcomes included size of ischemia demarcation in FLAIR sequence at 24 h; presence of cerebral microbleeds (CMB) according to established criteria at baseline [17]; presence of hemorrhagic transformation at 24 h according to the Heidelberg classification [18]; presence of more than one (multiple) acute ischemic lesions and severity of white matter hyperintensities according to the Fazekas score [19].

### Statistics

We compared the two OAC groups (DOAC versus VKA), and patients with confirmed and infra-therapeutic OAC using appropriate statistical measures (Fisher’s exact test for categorical variables, Mann-Whitney-U-Test for non-normally continuous or ordinally scaled variables, and Welch’s t-test for independent normally distributed data). We determined published predictors of stroke severity as pre-specified covariates and factors for the multivariate analysis avoiding collinearity. For the primary analysis, the association of OAC type (DOAC versus VKA) with any LVO or target LVO for EVT was assessed using binary logistic regression adjusting for the following confounders: age (continuous), sex (categorical), admission glucose (linear, adjusted odds ratio (aOR) per mmol/L increase), arterial hypertension (categorical) and atrial fibrillation (categorical). For the secondary analysis, the same confounders were included in a multivariable linear regression analysis. Patients with missing data items were excluded from the multivariate analysis. For the sensitivity analysis, the same model was used considering only patients with confirmed therapeutic OAC activity on admission. We used a level of significance of 0.05.

## Results

The random sample of OAC patients with AIS fulfilling the in- and exclusion criteria included 75 DOAC patients (59 Rivaroxaban, 12 Apixaban, 4 Dabigatran) and 61 VKA patients. Either documented information on compliance or a reliable assessment of DOAC activity was available in 45/75 (60%) of patients taking DOAC. INR was available in all patients taking VKA. OAC activity was confirmed therapeutic in 35/75 (46.7%) of DOAC patients and 45/61 (73.8%) of VKA patients. Table 1 summarizes the demographic and clinical data of the patients. Patients with preceding DOAC prescription were younger and had less severe white matter lesions as compared to VKA patients. Otherwise, groups were comparable for baseline and treatment variables.

**Table 1:** Baseline characteristics comparing patients with DOAC and VKA pretreatment

	DOAC pretreatment (***N*** = 75)	VKA pretreatment (***N*** = 61)	P	Confirmed therapeutic DOAC (***N*** = 35)	Confirmed therapeutic VKA (***N*** = 45)	P
Clinical items						
Age (years)	76 (67–82)	81 (75–84)	0.010	76 (68–83)	80 (74–84)	0.117
Body mass index (kg/m2)	26.4 (22.9–29.4)	25.8 (23.6–28.6)	0.674	27.0 (23.1–30.0)	25.7 (23.7–29.3)	0.376
Sex (female)	27/75 (36.0%)	26/61 (42.6%)	0.482	13/35 (37.1%)	20/45 (44.4%)	0.648
Preevent modified Rankin Scale	0.5 (0–1)	1 (0–2)	0.306	0 (0–1)	1 (0–2)	0.327
Onset			0.398			0.407
- Known	34/75 (45.3%)	36/61 (59.0%)		18/35 (51.4%)	29/45 (64.4%)	
- Wake-up	14/75 (18.7%)	7/61 (11.5%)		6/35 (17.1%)	4/45 (8.9%)	
- Unknown	27/75 (36.0%)	18/61 (29.5%)		11/35 (31.4%)	12/45 (26.7%)	
1st blood pressure, systolic	160 (136–176)	160 (146–182)	0.318	156 (135–178)	159 (143–176)	0.446
1st blood pressure, diastolic	79 (70–94)	88 (73–100)	0.089	78 (69–92)	87 (72–99)	0.145
1st Glucose (mmol/L)	6.2 (5.5–7.4)	6.5 (5.5–7.9)	0.500	6.3 (5.7–7.7)	6.6 (5.6–7.9)	0.773
1st Cholesterol, total (mmol/L)	4.4 (3.8–5.1)	4.5 (4.0–5.3)	0.244	4.5 (3.8–5.3)	4.5 (3.8–5.2)	0.848
1st Cholesterol, LDL (mmol/L)	2.4 (1.9–3.2)	2.4 (2.0–3.3)	0.570	2.4 (1.9–3.3)	2.4 (2.1–3.4)	0.587
1st Creatinine (umol/L)	87 (75–102)	88 (72–105)	0.666	89 (75–101)	86 (70–107)	0.809
NIHSS on admission	3 (2–8)	5 (1–10)	0.697	4.5 (2–8.5)	5 (1–12)	0.996
Medication						
Additional Antiplatelet	11/75 (14.7%)	11/61 (18.3%)	0.642	3/35 (8.6%)	9/45 (20%)	0.210
Lipid lowering drug	31/75 (41.3%)	26/61 (42.6%)	0.661	16/35 (45.7%)	22/45 (48.9%)	0.824
Anticoagulation						
- Confirmed therapeutic	35/75 (46.7%)	45/61 (73.8%)				
- Uncertain	30/75 (40%)	0				
- Confirmed non-therapeutic	10/75 (13.3%)	16/61 (26.2%)				
Risk factors						
Previous stroke	22/75 (29.3%)	14/61 (23.0%)	0.626	11/35 (31.4%)	9/45 (20%)	0.239
Previous transient ischemic attack	8/75 (10.7%)	7/61 (11.5%)	0.913	6/35 (17.1%)	6/45 (13.3%)	0.454
Arterial Hypertension	62/75 (82.7%)	53/61 (86.9%)	0.690	28/35 (90%)	40/45 (88.9%)	0.370
Diabetes	15/75 (20.0%)	13/61 (21.3%)	0.957	7/35 (20%)	9/45 (20%)	0.984
Hyperlipidemia	46/75 (61.3%)	41/61 (67.2%)	0.623	19/35 (54.3%)	30/45 (66.7%)	0.192
Smoking	15/75 (20.0%)	4/61 (6.6%)	0.073	7/35 (20%)	2/45 (4.4%)	0.076
Heart failure	11/75 (14.7%)	15/61 (24.6%)	0.328	5/35 (14.3%)	11/45 (24.4%)	0.296
Atrial Fibrillation	52/75 (69.3%)	41/61 (67.2%)	0.617	23/35 (65.7%)	31/45 (68.9%)	0.764
Mechanical Heart Valve	0	4/61 (6.6%)	0.098	0	3/45 (6.7%)	0.135
Low Ejection Fraction (< 30%)	2/75 (2.7%)	1/61 (1.6%)	0.657	1/35 (2.9%)	0	0.437
Peripheral artery disease	8/75 (10.7%)	4/61 (6.6%)	0.677	3/35 (8.6%)	4/45 (8.9%)	0.983
Treatment						
Acute recanalization therapy			0.905			0.464
- Intravenous thrombolysis	2/75 (2.7%)	1/61 (1.6%)		1/35 (2.9%)	0	
- Endovascular thrombectomy	10/75 (13.3%)	10/61 (16.4%)		3/35 (8.6%)	6/45 (13.3%)	
- Intraarterial thrombolysis	1/75 (1.3%)	1/61 (1.6%)		0	1/45 (2.2%)	
Imaging						
Fazekas Score	1 (0–2)	2 (1–3)	< 0.001	1 (0–2)	2 (1–3)	0.014
Cerebral Microbleeds present	28/72 (38.9%)	27/57 (47.4%)	0.373	16/33 (48.5%)	22/42 (52.4%)	0.818

*DOAC* Direct oral anticoagulant, *VKA* Vitamin K antagonist, *NIHSS* National Institute of Health Stroke Scale

There was no difference in the frequency of any LVO between DOAC and VKA patients (29.3% versus 37.7%, *P* = 0.361) on univariate analysis. There was also no difference in target LVO for EVT between DOAC and VKA patients (26.7% versus 27.9%, *P* = 1.0), but the size of the initial hypoperfusion was significantly larger in DOAC patients (80 mm, IQR 38–118 versus 49 mm, IQR 36–67; *P* = 0.039). The occlusion pattern was similar in patients with DOAC and VKA (Table 2) without a signal for more proximal or distal occlusions in either group. Equally, on binary logistic regression analysis DOAC as compared to VKA was not associated with the presence of any LVO (aOR 0.622, 95% CI 0.283–1.366) or presence of a target LVO for EVT (aOR 0.835, 95% CI 0.368–1.898).

**Table 2:** Patterns of Large Vessel Occlusions comparing patients with DOAC and VKA pretreatment

	DOAC pretreatment (***N*** = 75)	VKA pretreatment (***N*** = 61)	P	Confirmed therapeutic DOAC (***N*** = 35)	Confirmed therapeutic VKA (***N*** = 45)	P
Any large vessel occlusion	22/75 (29.3%)	23/61 (37.7%)	0.361	9/35 (25.7%)	14/45 (31.1%)	0.628
- Proximal ICA	1 (4.5%)	1 (4.3%)		0	0	
- Carotid-T	2 (9.1%)	2 (8.7%)		1 (11.1%)	1 (7.1%)	
- Proximal M1	7 (31.8%)	4 (17.4%)		3 (33.3%)	1 (7.1%)	
- Distal M1	3 (13.6%)	1 (4.3%)		1 (11.1%)	1 (7.1%)	
- M2	6 (27.3%)	6 (26.1%)		3 (33.3%)	4 (28.6%)	
- M3	0	1 (4.3%)		0	1 (7.1%)	
- A1	0	0		0	0	
- A2	1 (4.5%)	0		0	0	
- V4	0	1 (4.3%)		0	1 (7.1%)	
- BA	0	0		0	0	
- P1	0	1 (4.3%)		0	1 (7.1%)	
- P2/3	1 (4.5%)	4 (17.4%)		0	4 (28.6%)	
- Multiple	1 (4.5%)	1 (4.3%)		1 (11.1%)	0	
- SCA	0	1 (4.3%)		0	0	
Potential target large vessel occlusion for EVT	20/75 (26.7%)	17/61 (27.9%)	1.000	9/35 (25.7%)	9/45 (20.0%)	0.596

*DOAC* Direct oral anticoagulant, *VKA* Vitamin K antagonist, *ICA* Internal carotid artery, *M1* M1-segment of medial cerebral artery, *M2* M2-segment of medial cerebral artery, *M3* M3-segment of medial cerebral artery, *A1* A1-segment of anterior cerebral artery, *A2* A2-segment of anterior cerebral artery, *V4* V4-segment of vertebral artery, *BA* Basilar artery, *P1* P1-segment of posterior cerebral artery, *P2/3* P2 or P3-segment of posterior cerebral artery, *SCA* Superior cerebellar artery, *EVT* Endovascular stroke treatment

Sensitivity analysis considering only patients with confirmed therapeutic OAC did not change those results. The frequency of DWI/Perfusion mismatch was equal between groups. In those patients with any vessel occlusion, SWI could visualize the thrombus in 19/22 (86%) of DOAC patients (median 8 mm, IQR 6–20) and 57% of VKA patients (median 13 mm, IQR 7–24).

**Fig. 1:**
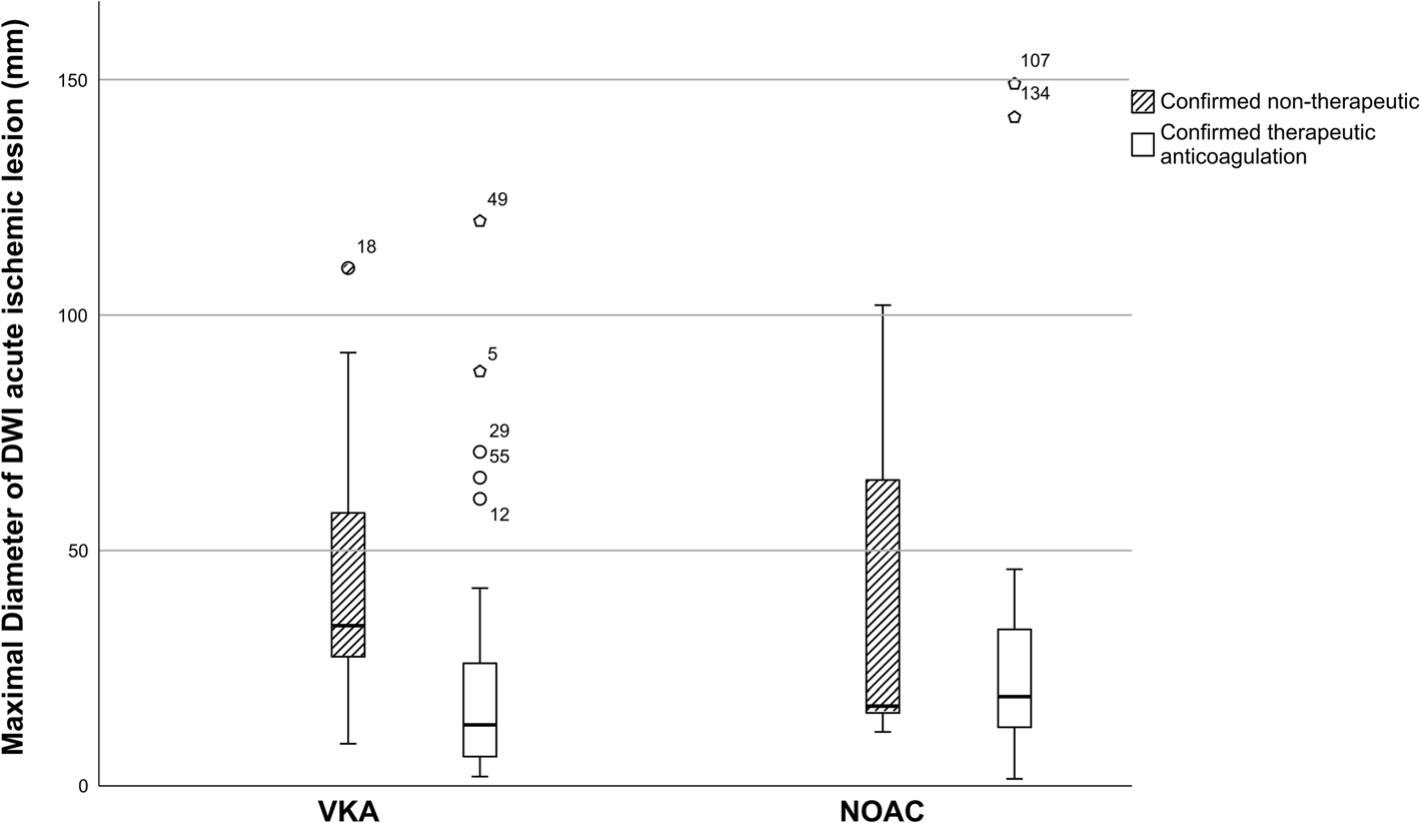
Lesion Diameter of Acute Ischemic Stroke according to confirmed versus non-therapeutic OAC according to strata of DOAC and VKA. Acute DWI lesion diameter in DOAC patients (median 18, IQR 11–36) as compared to VKA (median 20, IQR 7–36, *P* = 0.607). Lesion diameter in patients with VKA was significantly lower when OAC was therapeutic (median 13, IQR 6–26 versus median 20, IQR 7–36, *P* = 0.001 for Mann-Whitney-U-Test). NIHSS in patients with DOAC was equal when OAC was therapeutic (median 19, IQR 12–33 versus median 18, IQR 11–36, *P* = 0.705 for Mann-Whitney-U-Test)

For the secondary outcomes, maximal acute DWI lesion diameter in DOAC patients (median 18, IQR 11–36) was equal to VKA (median 20, IQR 7–36) on univariate analysis (*P* = 0.607, Table 3). Also, according to the multivariable linear regression analysis adjusting for confounders as outlined in the methods section, VKA was not significantly associated with increased acute DWI lesion diameter (β 2.385, 95%-CI -10.070 - 14.840, *P* = 0.705) as compared to DOAC. Both analyses remained unchanged when only considering patients with confirmed therapeutic OAC activity. There was no difference in the maximal diameter of the perpendicular axis of the acute ischemic lesion. In VKA patients, acute DWI lesion diameter was smaller in the case of confirmed therapeutic OAC activity. For DOAC patients, this effect could not be seen (Fig. 1).

**Table 3:** Outcome parameter comparing patients with DOAC and VKA pretreatment

	DOAC pretreatment (***N*** = 75)	VKA pretreatment (***N*** = 61)	P	Confirmed therapeutic DOAC (***N*** = 35)	Confirmed therapeutic VKA (***N*** = 45)	P
Clinical outcome						
NIHSS at 24 h	2 (1–4)	3 (0–7)	0.359	3 (1–5)	4 (0–8)	0.565
sICH ECASS-II	1/75 (1.3%)	1/61 (1.6%)	0.735	0	1/45 (2.2%)	0.298
Duration of hospital stay, days	4 (2–8)	4 (2–8)	0.345	4 (3–7)	4 (2–8)	0.359
90d mRS	1.5 (1–3)	3 (1.5–4)	0.001	1 (1–2)	3 (2–6)	< 0.001
90d recurrent stroke	2/75 (2.7%)	4/61 (6.6%)	0.255	0	3/45 (6.7%)	0.185
Imaging						
Diameter axis 1, initial DWI, mm	18 (11–36)	20 (7–36)	0.607	19 (12–33)	13 (6–26)	0.111
Diameter axis 2, initial DWI, mm	10 (6–19)	9 (5–23)	0.780	12 (6–19)	7 (4–16)	0.127
Diameter hypoperfusion axis 1, mm	80 (38–118), *N* = 40	49 (36–67), *N* = 37	0.039	83 (27–118), *N* = 16	49 (36–66), *N* = 13	0.079
Diameter hypoperfusion axis 2, mm	34 (18–40), N = 40	34 (14–40), N = 37	0.494	34 (20–46), N = 16	33 (13–38), N = 13	0.318
DWI/Perfusion Mismatch			0.443			0.714
- None	39/66 (59.1%)	29/53 (54.7%)		17/28 (60.7%)	26/40 (65.0%)	
- Small	4/66 (6.1%)	8/53 (15.1%)		1/28 (3.6%)	3/40 (7.5%)	
- Medium	4/66 (6.1%)	3/53 (5.7%)		2/28 (7.1%)	1/40 (2.5%)	
- Large	19/66 (28.8%)	13/53 (24.5%)		8/28 (28.6%)	10/40 (25%)	
Diameter axis 1, final FLAIR	18 (12–38)	27 (8–50)	0.409	18 (13–23)	20 (6–52)	0.945
Diameter axis 2, final FLAIR	10 (6–22)	14 (7–25)	0.397	10 (6–22)	11 (5–25)	0.908
Occlusion, any	22/75 (29.3%)	23/61 (37.7%)	0.361	9/35 (25.7%)	14/45 (31.1%)	0.628
Target occlusion for EVT	20/75 (26.7%)	17/61 (27.9%)	1.000	9/35 (25.7%)	9/45 (20.0%)	0.596
Thrombus length	8 (6–20), *N* = 19	13 (7–24), N = 13	0.388	15 (6–21), N = 7	11 (4–30), N = 6	0.836
Multiple Lesions	31/56 (55.4%)	24/49 (49.0%)	0.560	10/21 (47.6%)	20/41 (48.8%)	1.000
ICH, Heidelberg at follow up			0.024			0.126
- 1a	5/75 (6.7%)	3/61 (4.9%)		4/35 (11.4%)	3/45 (6.7%)	
- 1b	0	7/61 (11.5%)		0	5/45 (11.1%)	
- 2	0	1/61 (1.6%)		0	0	
- 3c	1/75 (1.3%)	0		1 (2.9%)	0	

*DOAC* Direct oral anticoagulant, *VKA* Vitamin K antagonist, *NIHSS* National Institute of Health Stroke Scale, *sICH ECASS II* symptomatic intracranial hemorrhage according to the European Co-operative Acute Stroke Study-II definition, *mRS* modified Rankin Scale, *DWI* Diffusion weighted imaging, *FLAIR* Fluid attenuated inversion recovery sequence, *EVT* Endovascular stroke treatment, *ICH* Intracranial hemorrhage

Size of ischemia lesion diameter at 24 h (FLAIR sequence) was equal in DOAC patients (median 18 mm, IQR 12–38) and VKA patients (median 27 mm, IQR 8–50, *P* = 0.409). The rate of multiple lesions was equal between groups.

At baseline, 38.9% of DOAC patients and 47.4% of VKA patients had at least one CMB. One patient (1.3%) in the DOAC group (Heidelberg class 3) and one patient (1.6%) in the VKA group (Heidelberg class 2) suffered symptomatic intracranial hemorrhage at 24 h. 5/75 (6.7%) of DOAC patients and 10/61 (11.5%) of VKA patients showed asymptomatic hemorrhagic transformation of the ischemic lesion at 24 h.

## Discussion

The comparison of MRI findings in patients with AIS and preceding DOAC versus VKA prescription in our real world university dataset shows the following main findings:

(1) Between DOAC and VKA patients, the frequencies of any LVO (29.3% versus 37.7%, *P* = 0.361), and target LVO for endovascular therapy (26.7% versus 27.9%, *P* = 1.0; aOR 0.835, 95% CI 0.368–1.898) were equal with a similar occlusion pattern. (2) Also, the presence of multiple lesions and thrombus characteristics were similar in DOAC and VKA patients. (3) Ischemic lesion diameter in real world patients is equal in patients taking DOAC and VKA and this finding holds true in case of confirmed therapeutic OAC activity. (4) Lesion size in VKA patients was smaller in the setting of confirmed therapeutic VKA. (5) The frequency of radiological hemorrhagic transformation and symptomatic intracranial hemorrhage in OAC patients was low.

AIS in the setting of OAC accounts for about 10% of all AIS in comprehensive stroke centers with rapidly increasing numbers of preceding DOAC prescriptions due to the increasing number of indications [20, 21]. Besides providing an accurate diagnosis, neuroimaging aids clinicians in patient selection for IVT and EVT as well as early medical management, prognosis assessment and can provide clues to the etiology of the event and hence guide etiological work-up.

Our data show that about 1 in 4 patients with preceding DOAC or VKA has a target LVO for EVT. Macha et al. reported similar frequencies with a decreasing rate of LVO with increasing DOAC plasma levels [7]. Fittingly, Woo et al. reported a LVO rate of 33% in their cohort of 120 AIS patients with preceding DOAC therapy [8]. The similar occlusion patterns, clot burden and frequency of multiple lesions provide insights into the mechanisms associated with OAC failure and argue for similar stroke etiologies and pathophysiological mechanisms of thrombus formation despite different pharmacological modes of action of preceding VKA and DOAC therapy. In both DOAC and VKA patients, SWI could visualize the thrombus with a higher rate in DOAC (86%) as compared to VKA patients (57%). However, due to the small number of patients, this difference in sensitivity to detect the thrombus might be by chance.

Sakamoto et al. found DOAC therapy to be associated with smaller infarct sizes on MRI in a smaller study (40 non-therapeutic VKA, 22 confirmed therapeutic VKA and 29 DOAC, 239 controls) [9]. However, the authors compared non-therapeutic VKA and DOAC treatment with controls without laboratory assessment of DOAC activity or information on compliance. Importantly, the size of infarction for sufficient VKA and DOAC was comparable in their cohort. Oguro et al. also reported smaller infarction sizes in a case series of 7 VKA and 10 DOAC patients with recurrent AIS and also found reduced ischemia volume with DOAC pretreatment [22]. This study was limited by the small sample size and the univariate analysis despite marked baseline differences in CHA2DS2–VASc score, stroke severity of index event and renal dysfunction. Our main finding of equal acute and final infarction diameter in DOAC as compared to VKA patients fits to recently published data on equal clinical stroke severity of patients with preceding DOAC and VKA therapy in real-world patients [16]. Furthermore, the reduced lesion diameter observed in our study in the setting of confirmed VKA therapy is in line with previous findings of smaller ischemic lesions [23] and reduced clinical stroke severity when VKA therapy is within the target range [24]. Macha et al. reported reduced stroke severity in the setting of confirmed DOAC therapy [7]; however our study might have lacked power to prove this difference also in cerebral lesion size.

The low rate of radiological as well as clinically symptomatic intracranial hemorrhage is reassuring; however recanalization strategies were very infrequently used in this cohort.

### Strengths and limitations

The data was collected prospectively, but our study has the inherent limitations of a single-center retrospective analysis. We reliably differentiated between therapeutic and non-therapeutic OAC in patients prescribed VKA and DOAC. For assessment of lesion size, we only measured the diameter and did not use three-dimensional segmentation tools, which does not accurately reflect true ischemic lesion size. Nevertheless, the correlation between maximal diameter and volume seems acceptable [25] and our main focus was the presence of LVO. The cutoff of 1/3 used in the mismatch analysis was arbitrary and might not be easily comparable to more commonly used cutoffs such as > 20% of the ischemic lesion. Although baseline characteristics of the DOAC and VKA group were almost identical, we might have missed confounding baseline variables influencing the physicians’ indication of DOAC/VKA and affecting imaging outcomes. Due to the limited number of patients with DOAC other than Rivaroxaban, our findings should not be extrapolated to those substances. Therefore, the findings have to be replicated in further cohorts.

## Conclusion

In conclusion, we found that about 1 in 4 patients with preceding OAC has a target LVO for EVT. The ischemic lesion size was equal in patients taking DOAC and VKA, also in the setting of confirmed therapeutic OAC activity. Since imaging features are similar, the mechanisms associated with AIS despite preceding DOAC or VKA treatment seem alike.

## Data Availability

The datasets used and/or analysed during the current study are available from the corresponding author on reasonable request.

## Abbreviations

ADC: Apparent Diffusion Coefficient
AF: Atrial Fibrillation
AIS: Acute Ischemic stroke
aOR: adjusted Odds Ratio
CMB: Cerebral Microbleeds
DOAC: Direct Oral Anticoagulants
DWI: Diffusion-Weighted Imaging
DSC: Dynamic Susceptibility Contrast
EVT: Endovascular Therapy
FLAIR: Fluid-Attenuated Inversion Recovery
INR: International Normalized Ratio
IVT: Intravenous Thrombolysis
LVO: Large Vessel Occlusion
MRI: Magnetic Resonance Imaging
NIHSS: National Institutes of Health Stroke Scale
OAC: Oral Anticoagulation
SWI: Susceptibility-Weighted Imaging
TOF: Time of Flight
VKA: Vitamin K Antagonists

## References

[1] Kirchhof P, Benussi S, Kotecha D, Ahlsson A, Atar D, Casadei B (2016). 2016 ESC guidelines for the management of atrial fibrillation developed in collaboration with EACTS. Eur Heart J.

[2] Ruff CT, Giugliano RP, Braunwald E, Hoffman EB, Deenadayalu N, Ezekowitz MD (2014). Comparison of the efficacy and safety of new oral anticoagulants with warfarin in patients with atrial fibrillation: a meta-analysis of randomised trials. Lancet..

[3] Almutairi AR, Zhou L, Gellad WF, Lee JK, Slack MK, Martin JR (2017). Effectiveness and safety of non-vitamin K antagonist oral anticoagulants for atrial fibrillation and venous thromboembolism: a systematic review and meta-analyses. Clin Ther.

[4] El-Koussy M, Schroth G, Brekenfeld C, Arnold M (2014). Imaging of acute ischemic stroke. Eur Neurol.

[5] Menon BK, Campbell BCV, Levi C, Goyal M (2015). Role of imaging in current acute ischemic stroke workflow for endovascular therapy. Stroke..

[6] Tong Elizabeth, Hou Qinghua, Fiebach Jochen B., Wintermark Max (2014). The role of imaging in acute ischemic stroke. Neurosurgical Focus.

[7] Macha Kosmas, Marsch Armin, Siedler Gabriela, Breuer Lorenz, Strasser Erwin F., Engelhorn Tobias, Schwab Stefan, Kallmünzer Bernd (2019). Cerebral Ischemia in Patients on Direct Oral Anticoagulants. Stroke.

[8] Woo HG, Chung I, Gwak DS, Kim BK, Kim BJ, Bae HJ (2019). Recurrent ischemic stroke in atrial fibrillation with non-vitamin K antagonist oral anticoagulation. J Clin Neurosci.

[9] Sakamoto Y, Okubo S, Sekine T, Nito C, Suda S, Matsumoto N (2018). Prior direct Oral anticoagulant therapy is related to small infarct volume and no major artery occlusion in patients with stroke and non-Valvular atrial fibrillation. J Am Heart Assoc.

[10] Kanai Y, Oguro H, Tahara N, Matsuda H, Takayoshi H, Mitaki S (2018). Analysis of recurrent stroke volume and prognosis between warfarin and four non–vitamin K antagonist Oral anticoagulants’ Administration for Secondary Prevention of stroke. J Stroke Cerebrovasc Dis.

[11] Lim MS, Chapman K, Swanepoel P, Enjeti AK (2016). Sensitivity of routine coagulation assays to direct oral anticoagulants: patient samples versus commercial drug-specific calibrators. Pathology..

[12] Ten Cate H, Henskens YM, Lancé MD (2017). Practical guidance on the use of laboratory testing in the management of bleeding in patients receiving direct oral anticoagulants. Vasc Health Risk Manag.

[13] Toyoda K, Yamagami H, Koga M (2018). Consensus guides on stroke thrombolysis for Anticoagulated patients from Japan: application to other populations. J stroke.

[14] Douxfils J, Ageno W, Samama CM, Lessire S, ten Cate H, Verhamme P (2018). Laboratory testing in patients treated with direct oral anticoagulants: a practical guide for clinicians. J Thromb Haemost.

[15] Touzé E, Gruel Y, Gouin-Thibault I, De Maistre E, Susen S, Sie P (2018). Intravenous thrombolysis for acute ischemic stroke in patients on direct oral anticoagulants. Eur J Neurol.

[16] Auer E, Frey S, Kaesmacher J, Hakim A, Seiffge DJ, Goeldlin M (2019). Stroke severity in patients with preceding direct oral anticoagulant therapy as compared to vitamin K antagonists. J Neurol.

[17] Greenberg SM, Vernooij MW, Cordonnier C, Viswanathan A, Al-Shahi Salman R, Warach S (2009). Cerebral microbleeds: a guide to detection and interpretation. Lancet Neurol.

[18] Von Kummer R, Broderick JP, Campbell BCV, Demchuk A, Goyal M, Hill MD (2015). The Heidelberg bleeding classification: classification of bleeding events after ischemic stroke and reperfusion therapy. Stroke..

[19] Wahlund LO, Barkhof F, Fazekas F, Bronge L, Augustin M, Sjögren M (2001). A new rating scale for age-related white matter changes applicable to MRI and CT. Stroke..

[20] Meinel TR, Kaesmacher J, Chaloulos-Iakovidis P, Panos L, Mordasini P, Gralla J, Fischer U (2019). Clinical effect of successful reperfusion in patients presenting with posterior circulation large vessel occlusion: Data from a multicenter registry.

[21] Seiffge DJ, Kägi G, Michel P, Fischer U, Béjot Y, Wegener S (2018). Rivaroxaban plasma levels in acute ischemic stroke and intracerebral hemorrhage. Ann Neurol.

[22] Kanai Yukie, Oguro Hiroaki, Tahara Nao, Matsuda Hanako, Takayoshi Hiroyuki, Mitaki Shingo, Onoda Keiichi, Yamaguchi Shuhei (2018). Analysis of Recurrent Stroke Volume and Prognosis between Warfarin and Four Non–Vitamin K Antagonist Oral Anticoagulants' Administration for Secondary Prevention of Stroke. Journal of Stroke and Cerebrovascular Diseases.

[23] Ay H, Arsava EM, Gungor L, Greer D, Singhal AB, Furie KL (2008). Admission international normalized ratio and acute infarct volume in ischemic stroke. Ann Neurol.

[24] Hylek EM, Go AS, Chang Y, Jensvold NG, Henault LE, Selby JV (2003). Effect of intensity of Oral anticoagulation on stroke severity and mortality in atrial fibrillation. N Engl J Med.

[25] Gillard Jonathan H., Waldman Adam D., Barker Peter B. (2009). Clinical MR Neuroimaging.

